# Novel insights into the somatic proteome of *Strongyloides stercoralis* infective third-stage larvae

**DOI:** 10.1186/s13071-023-05675-7

**Published:** 2023-01-31

**Authors:** Klevia Dishnica, Chiara Piubelli, Marcello Manfredi, Ravi Teja Kondaveeti, Silvia Stefania Longoni, Monica Degani, Dora Buonfrate, Alejandro Giorgetti, Natalia Tiberti

**Affiliations:** 1 Department of Biotechnology, University of Verona, Verona, Italy; 2 Department of Infectious, Tropical Diseases and Microbiology, IRCCS Sacro Cuore Don Calabria Hospital, Negrar Di Valpolicella, Italy; 3 Department of Translational Medicine, University of Piemonte Orientale, Novara, Italy

**Keywords:** *Strongyloides stercoralis*, iL3, Proteomics, Annotation, B-cell epitope prediction, Serodiagnosis

## Abstract

**Background:**

Strongyloidiasis is a neglected tropical disease affecting an estimated 600 million people, particularly in resource-limited settings. The infection can persist lifelong due to unusual auto-infective cycle of *Strongyloides stercoralis*. The lack of a diagnostic gold standard and limited knowledge of the mechanisms underpinning this chronic infection are key issues in disease management. To date, only a few proteomics studies have been conducted to elucidate the molecular mechanisms associated with *Strongyloides* parasitism or to highlight novel immunological markers, with the result that our knowledge of *S. stercoralis* proteome remains limited. This study aims at expanding the characterization of *S. stercoralis* infective larvae (iL3) in order to further explore the mechanisms of parasitism and to highlight possible novel targets for serodiagnosis.

**Methods:**

iL3 obtained from an infected subject were analysed by high-throughput tandem mass spectrometry. To achieve a more comprehensive characterization of the iL3 proteome we analysed the experimental dataset using an automatic search strategy combined with manual annotation, which included gene ontology (GO) analysis, InterPro annotation, assessment of the homology with *Homo sapiens* and other pathogens of clinical importance and B-cell epitope prediction.

**Results:**

Our pipeline identified 430 *S. stercoralis* proteins, 187 (43%) of which were uncharacterized. Oxidoreductases and peptidases were amongst the most represented protein categories, as highlighted by molecular function GO analyses, while membrane and mitochondrial proteins were the most represented cellular component GO categories. A high proportion of proteins bearing the CAP, SCP or thioredoxin domain or belonging to cysteine-rich secretory, transthyretin-like or peptidase protein families were also identified. Additionally, we highlighted nine proteins displaying low homology with *H. sapiens* or other related pathogens and bearing amino acid sequences with immunogenic properties.

**Conclusions:**

Our comprehensive description and annotation of the *S. stercoralis* iL3 proteome contribute to expanding the ‘omics characterization of this parasite and provide experimental evidence on the most represented proteins associated with *S. stercoralis* parasitism, as inferred from genomic and transcriptomic data. Moreover, novel candidate immunogenic proteins to be evaluated as novel serological diagnostic markers are highlighted.

**Graphical Abstract:**

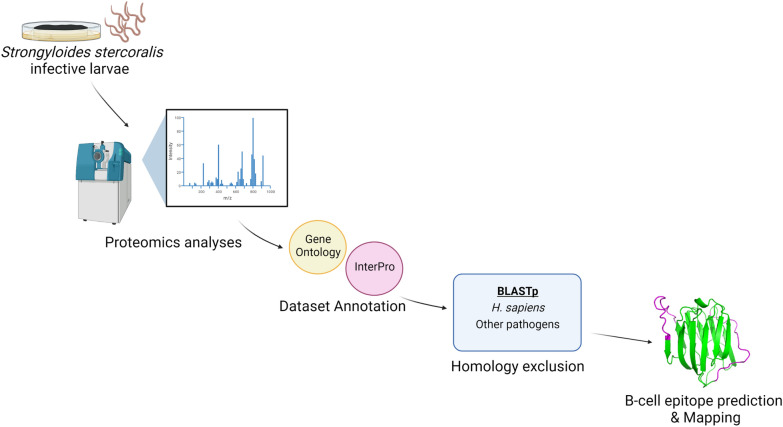

**Supplementary Information:**

The online version contains supplementary material available at 10.1186/s13071-023-05675-7.

## Background

Human strongyloidiasis caused by *Strongyloides stercoralis* is a soil-transmitted helminthiasis that has recently been listed by the WHO among the tropical neglected diseases requiring control actions in endemic areas [1]. Strongyloidiasis is estimated to affect about 600 million people worldwide [2], mostly in tropical and subtropical regions. However, foci of autochthonous strongyloidiasis have also been reported in temperate areas, including Italy, Spain, Japan, Australia and USA [3]. *Strongyloides stercoralis* belongs to the phylum Nematoda, clade IV [4]. Its life-cycle is complex, alternating between cycles of free-living and parasitic stages. Humans acquire the infection through the penetration of the intact skin by infective filariform larvae (iL3) present in contaminated soil which, once in the host, migrate through different organs. During migration, the larvae moult until they become adult worms, which ultimately settle in the small intestine. Once there, the parthenogenetic females deposit eggs that hatch in rhabditiform larvae (L1), which are then excreted in stools and initiate the free-living cycle. However, some L1 undergo an auto-infective cycle, i.e. mature into invasive filariform larvae, in the large intestine and penetrate the intestinal mucosa or the perianal skin to continue the parasitic life-cycle. This  peculiar life-cycle allows *S. stercoralis* to perpetuate the infection, in the absence of treatment, potentially indefinitely [5].

In immunocompetent subjects, the infection mostly leads to a chronic indolent condition; however, changes in the host immune status can cause a dramatic increase in parasite burden, known as hyper-infection or dissemination, which can be life-threatening [5].

The diagnosis of strongyloidiasis is challenging, with most available methods presenting variable sensitivity [5, 6]. The most sensitive diagnostic tools are serological immunoassays [7, 8]. Most commercial assays are based on crude larval antigens, which reduce their specificity and result in a high batch-to-batch variability. The development of assays based on recombinant antigens represents a very promising strategy to avoid the need of constant supply of parasites, to overcome the variability of the antigenic source and to reduce cross reactions with other helminths, factors that affect the performance of current serological tests [5]. Indeed, a novel commercial enzyme-linked immunosorbent assay (ELISA) based on the detection of two recombinant antigens, Ss-NIE and Ss-IR, has recently been developed and evaluated on cryopreserved samples. The test has shown variable accuracy in two different studies, probably due to the lack of a diagnostic gold standard, and has yet to be tested prospectively. Nonetheless, at present it is among the most sensitive and specific serological tests for strongyloidiasis, further highlighting the potential of recombinant antigens for serodiagnosis [9, 10].

To date, only a few proteomics studies have been conducted to elucidate the molecular mechanisms associated with *Strongyloides* parasitism or to highlight novel immunological markers [11–17]. Consequently, our knowledge of *S. stercoralis* proteome is still limited. In order to highlight novel targets to improve current serodiagnosis and treatment, it is fundamental to expand the molecular understanding derived from ‘omics studies beyond the current state of the art. Indeed, an in depth characterization of the *S. stercoralis* infective larvae proteome might reveal on one hand novel players in the mechanisms of host–pathogen interaction and on the other hand potentially immunogenic proteins to be used for the development of novel diagnostic serological tests, as well as target proteins for new therapeutics.

The aim of this study was to expand the characterization of *S. stercoralis* iL3 proteome as established by high-throughput proteomics, combining automatic search strategies and manual annotation.

## Methods

### Larvae isolation, protein extraction and digestion

*Strongyloides stercoralis* larvae were obtained from a human subject. Fresh stools mixed with charcoal and saline were cultivated using the agar plate culture method . iL3 larvae were harvested after 3 days of culture [18], concentrated by centrifugation and incubated with phosphate buffered saline (PBS) supplemented with 100 U/ml penicillin, 100 µg/ml streptomycin and 0.625 µg/ml amphotericin B (all from Gibco, Thermo Fisher Scientific, Waltham, MA, USA) for 2 h at 4 °C. Larvae were then washed twice with cold PBS, counted under the microscope and stored at − 80 °C for future use.

A pellet of 10,000 iL3 was re-suspended in 0.1% RapiGest SF (Waters Corporation, Milford, MA, USA) in 0.1 M triethylammonium bicarbonate buffer (TEAB) pH 8.0, sonicated with breaks on ice and incubated for 10 min at 80 °C, following a protocol reported in [19]. The sample was then centrifuged at 14,000 *g* for 10 min at 4 °C and the supernatant recovered. Protein concentration was determined by the Qubit protein assay (Life Technologies, Thermo Fisher Scientific). A 75-µg sample of proteins was reduced with 50 mM Tris-(2-carboxyethyl)phosphine hydrochloride (TCEP), alkylated with 15 mM iodoacetamide and digested with 0.25 μg/μl sequencing grade-modified trypsin (Roche, Basel, Switzerland; 1:25 protease to protein ratio). The sample was incubated with 1% trifluoroacetic acid for 45 min at 37 °C to cleave the RapiGest SF surfactant, cleaned with C18 spin columns (Pierce™, Thermo Fisher Scientific) and dried under vacuum prior to liquid chromatography-tandem mass spectrometry (LC–MS/MS) analyses.

### Protein identification by LC–MS/MS

Trypsin-digested protein samples were analysed with a micro-LC system (Eksigent Technologies, Dublin, CA, USA) coupled with the TripleTOF 5600+ system (Sciex, Concord, ON, Canada) equipped with a DuoSpray ion source (Sciex). The stationary phase was a Halo C18 column (0.5 × 100 mm, 2.7 µm; Eksigent Technologies). The mobile phase was a mixture of 0.1% (v/v) formic acid in water (phase A) and 0.1% (v/v) formic acid in acetonitrile (phase B), eluting at a flow rate of 15.0 µl/min at an increasing concentration of solvent B from 2% to 40% in 30 min. Samples were also analysed with nano liquid chromatography using an Acclaim PepMap C18 column 2 μm, 75 µm × 150 mm (Thermo Fisher Scientific) and injection volume of 2 μl. The flow rate was 300 nl/min, phase A was 0.1% formic acid/water and phase B was 80% acetonitrile/0.1% formic acid/20% water. A 2-h gradient was used (3–45%). Identification was performed using a data-dependent acquisition (DDA) method: the MS analysis was carried out using a mass range of 100–1500 Da (time-of-flight scan with an accumulation time of 0.25 s), followed by a MS/MS product ion scan from 200 to 1250 Da (accumulation time of 5.0 ms) with the abundance threshold set at 30 cps (35 candidate ions can be monitored during each cycle) [20]. The MS data were acquired with Analyst TF 1.7 (Sciex). The DDA files were searched using Protein Pilot software v. 4.2 (Sciex) and Mascot v. 2.4 (Matrix Science Inc., Boston, MA, USA) using trypsin as the enzyme, with two missed cleavages, a search tolerance of 50 ppm for the peptide mass tolerance and 0.1 Da for the MS/MS tolerance [21]. Searches were performed using the UniProt Swiss-Prot database for *Strongyloides stercoralis* (version 01/02/2020, taxon: 6248, proteome ID: UP000035681, protein count: 12,978), with a false discovery rate (FDR) fixed at 1%.

The MS proteomics data have been deposited in the ProteomeXchange Consortium via the PRIDE [22] partner repository with the dataset identifier PXD037243.

### Bioinformatics analyses

**Fig. 1:**
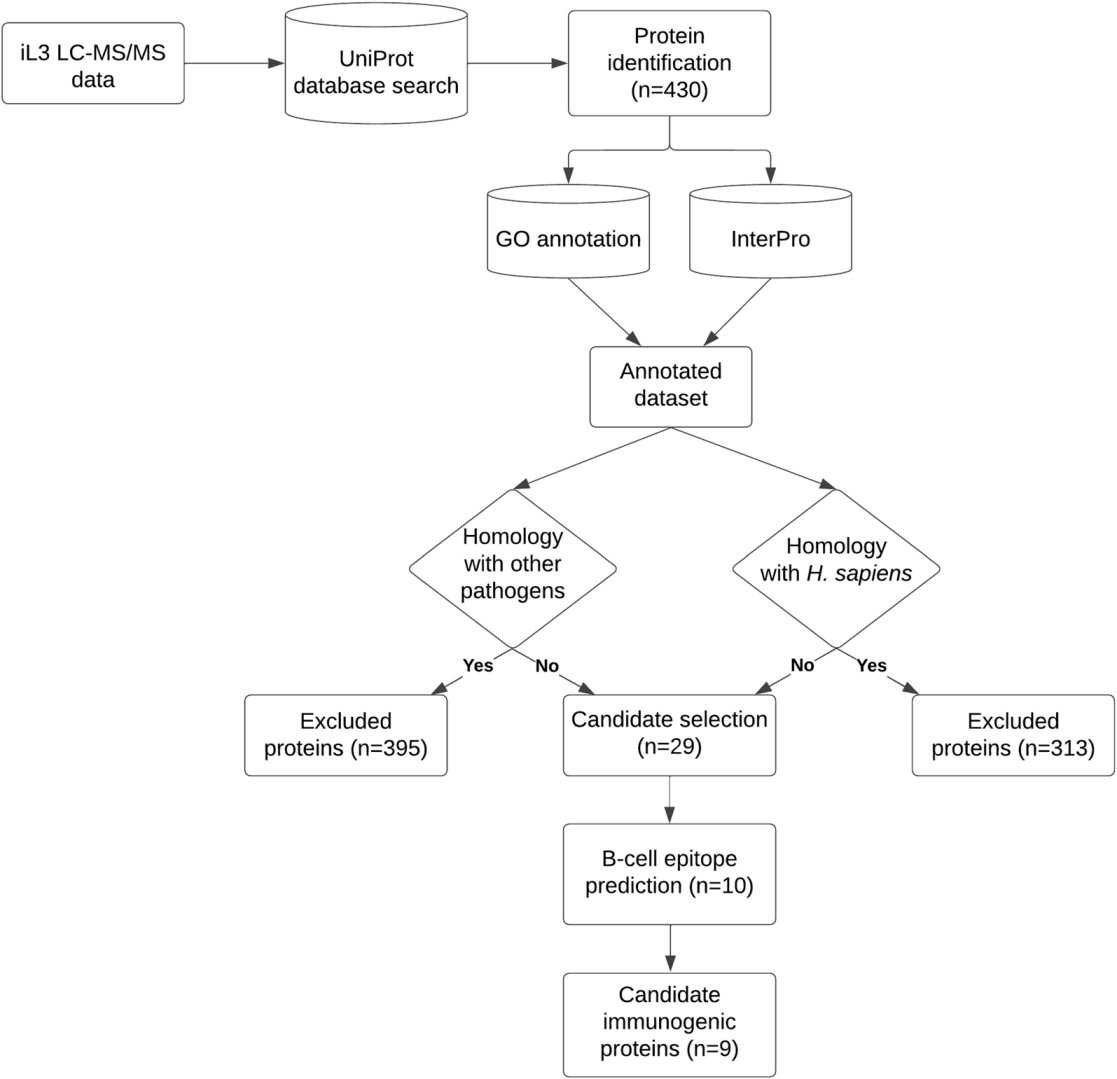
Study flowchart. Pipeline followed in the present study. GO, Gene ontology; iL3, infective filariform larvae; LC-MS/MS, Liquid chromatography-tandem mass spectrometry. Other pathogens include: *Ancylostoma duodenale; Ancylostoma ceylanicum; Necator americanus; Ascaris lumbricoides; Trichuris trichiura; Toxocara canis; Loa loa; Mansonella perstans; Mansonella ozzardi; Wuchereria bancrofti; Onchocerca volvulus; Brugia malayi; Brugia timori; Dirofilaria immitis; Dirofilaria repens; Trichinella spiralis; Taenia saginata; Taenia solium; Echinococcus granulosus; Hymenolepis nana; Schistosoma mansoni; Schistosoma haematobium; Schistosoma japonicum; Fasciola hepatica; Plasmodium falciparum; Plasmodium vivax; Plasmodium ovale; Plasmodium malariae; Plasmodium knowlesi*

The bioinformatics analyses were carried out by automatizing a standard protocol for the annotation. The protocol included: (i) a BLASTp [23] search using default parameters against Uniprot 2022 [24]; (ii) each protein identifier was then connected with its associated GO terms [25] via UniProt and GO terms were then organized using QuickGO tool [26]; and (iii) proteins were then classified into families or domains, and important sites were predicted, retrieving this information by InterPro [27]. In order to identify possible candidates for immunogenic epitope prediction, the annotated dataset of proteins was then investigated using BLASTp for homology with the human protein database and with a list encompassing 29 clinically relevant pathogens (24 helminths and 5 *Plasmodium* spp.) that might co-infect individuals with strongyloidiasis (Fig. 1). The threshold for considering an *S. stercoralis* protein as having low homology with proteins of human or  with those of other pathogens' origin was empirically established based on the BLASTp e-value obtained for the L3NieAg.01 (AC: Q9UA16), which is known to have a good specificity when used in serodiagnosis [9]. Thus, a BLASTp e-value threshold of 4E-25 and 2E-30 was applied for the comparison with *H. sapiens* or with other pathogens, respectively.

Linear B-cell epitopes were predicted from protein sequences using the different web-based tools available via the Immune Epitope Database Analysis Resource (IEDB; available at http://tools.iedb.org/main/). The following physicochemical properties of individual residues were explored and scored: beta-turn, surface accessibility, antigenicity and hydrophilicity, as already reported in the literature [28]. All residues having an individual score equal or higher than the average protein score were highlighted. In parallel, prediction was also performed using BepiPred-2.0, which combines a hidden Markov model (HMM) with an amino acid propensity scale [29]. Proteins of potential interest for bearing B-cell epitopes were then manually analysed and selected based on the following parameters: (i) sequences of at least 8 amino acids; (ii) a BepiPred-2.0 score > 0.5 (range 0–1); and (iii) at least three physicochemical properties above their thresholds (calculated as the mean of the scores of all individual residues). The specific sequences of interest were highlighted and visualized in the proteins three-dimensional model using Pymol v2.4.1 [30]. Due to the lack of structural characterization of *S. stercoralis* proteins on Protein Data Bank (PDB) [31], selected proteins of interest containing predicted epitopes were structurally predicted using AlphaFold [32].

## Results and discussion

In the present study, we analysed the proteome of *S. stercoralis* infective larvae by LC-MS/MS and performed a semi-automated annotation of the dataset to achieve a more in depth characterization of larval proteome and to predict potential immunogenic proteins of interest for the development of new sero-diagnostic tools. The study flowchart is reported in Fig. 1. Our high-throughput MS analysis identified 430 proteins (2 unique peptides, 1% FDR), which to the best of our knowledge is the largest experimental proteome of *S. stercoralis* iL3 reported to date (Additional file 1: Table S1). Indeed, only one study had previously employed untargeted proteomics to investigate the *S. stercoralis* iL3 proteome; however, due to the lack of a reference genome at that time, only 26 proteins were identified [12].

A high-quality draft genome of *S. stercoralis* was assembled in 2016 (42.6 Mb) and predicted to contain 13,098 protein-coding genes [11], facilitating the annotation of ‘omics data, although a reference genome has yet to be assembled. Hunt et al*.* performed an in depth investigation of the genomic bases of parasitism in the *Strongyloides* clade by comparing distinct life stages of different *Strongyloides* species, the closely related *Parastrogyloides trichosuri* and the free-living *Rhabditophanes* at the genome, transcriptome and proteome level [11]. This comparison has allowed researchers to propose protein categories with a putative role in parasitism that are expanded in the *S. stercoralis* genome or abundantly transcribed in iL3. These include proteinases (astacins—metallopeptidases, aspartic proteases, prolyl oligopeptidase), protease inhibitors, SCP/TAPS proteins, transthyretin-like proteins and acetylcholinesterases [11, 33]. Interestingly, the same protein families were also identified in *Strongyloides venezuelensis* iL3 [15]. Similarly, next generation RNA sequencing was employed to evaluate the association between larval development in an *S. stercoralis* laboratory strain (i.e. PV001) and the expression of specific genes homologous of *Caenorhabditis elegans*, in which they were reported to be involved in dauer arrest or activation [34].

**Fig. 2:**
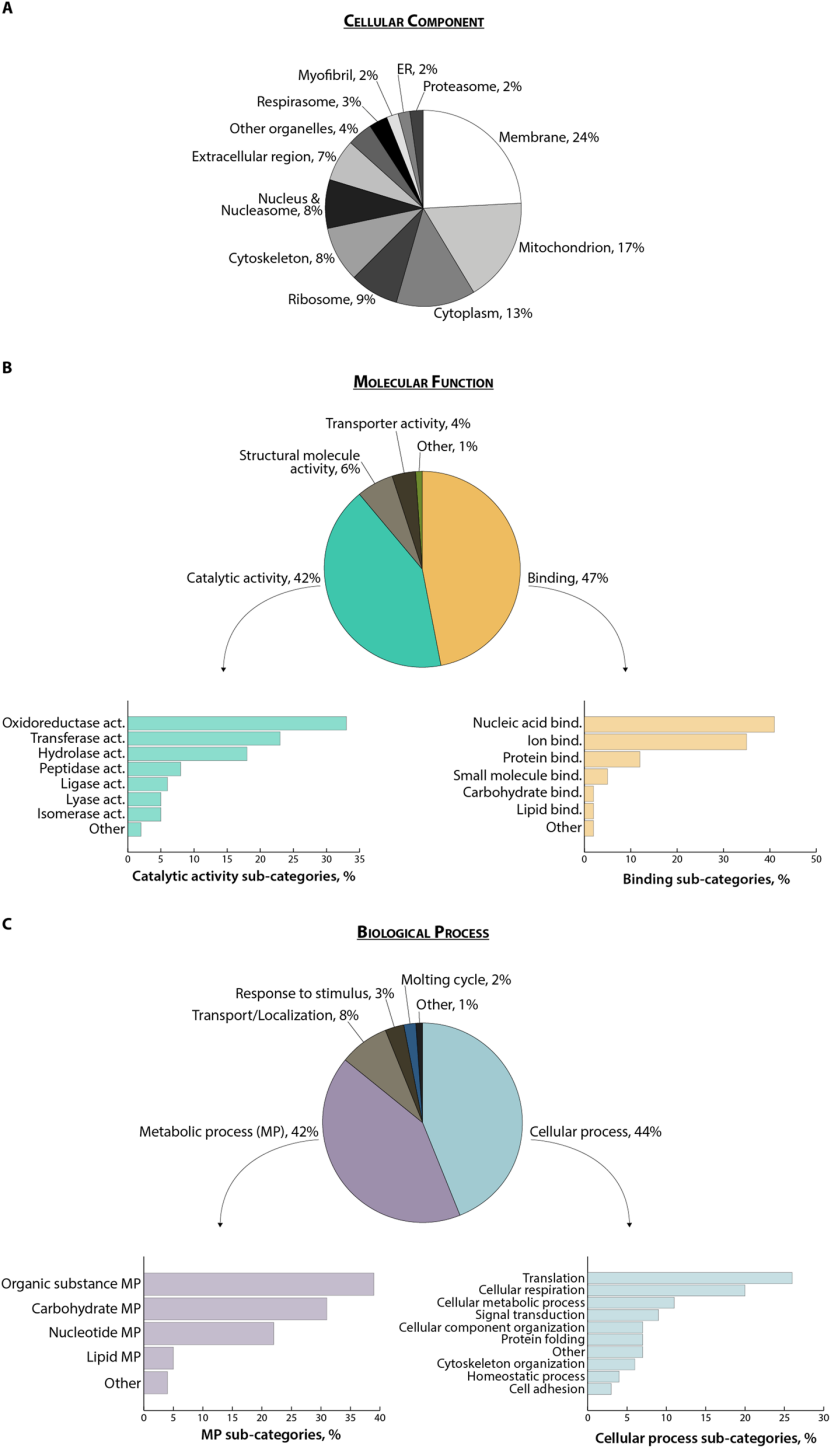
Gene ontology results. Frequency of the GO terms for the three categories cellular component (**A**), molecular function (**B**) and biological process (**C**) across identified proteins. ER, Endoplasmic reticulum

**Fig. 3:**
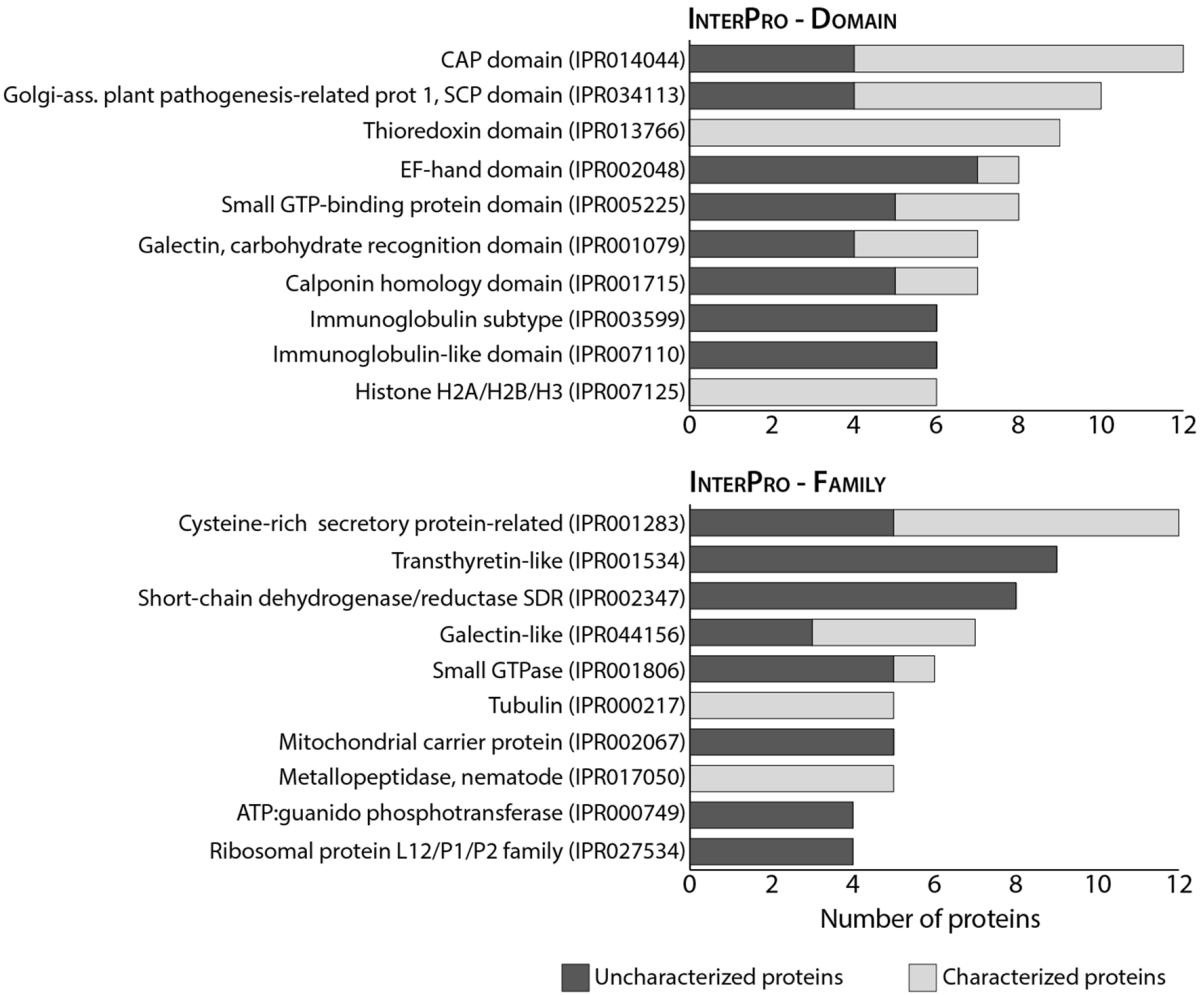
InterPro annotation results. The top 10 most frequent InterPro terms for the categories domain and family represented among all identified proteins (*n* = 430), as established by InterPro annotation. For each term, the number of uncharacterized and characterized proteins is represented in different color shades. The complete annotation is reported in Additional file 1: Table S1

In our dataset, 43% of the identified protein sequences (i.e. 187 protein matches) corresponded to uncharacterized proteins according to UniProt database 2022 for *Strongyloides stercoralis*. In order to achieve a better characterization of the dataset we performed a semi-automated annotation through GO and InterPro functional analyses (Additional file 1: Table S1). The cellular component (CC) GO analysis highlighted a prevalence of membrane and mitochondrial proteins, which together accounted for > 40% of the annotated terms (Fig. 2a). Almost half (47%) of the molecular function (MF) GO terms had binding activities, with nucleic acid and nucleotide binding being the most represented sub-categories, while 40% were associated with enzymatic activities (Fig. 2b). Intriguingly, within this latter group, the most represented term corresponded to oxidoreductase activity, accounting for 33% of all GO terms associated with catalytic activities. It could be speculated that infective larvae might need to counteract the oxidative stress either derived from their particularly active cellular metabolism or as a defence mechanism against the host immune response [35, 36]. Interestingly, we identified three of the four major antioxidant enzyme families involved in the response against reactive oxygen species (ROS), namely glutathione peroxidase, superoxide dismutase and peroxiredoxin/thioredoxin. Antioxidant enzymes are known to be important in nematodes, and an evolutionary analysis has been recently published [35]. Antioxidative enzymes were also identified in the excretory-secretory products (ESPs) from the different life stages of *Strongyloides ratti* [14] and *S. venezuelensis* iL3 [17]; it would be interesting to evaluate whether the expression of these proteins is modulated during parasite development. The biological process (BP) GO analysis also highlighted that *S. stercoralis* iL3 larvae express a high number of proteins involved in metabolic (42%) and cellular processes (44%) (Fig. 2c). Notably, the most represented cellular processes were translation and cellular respiration, which is in agreement with the high number of nucleic acid/nucleotide binding proteins and mitochondrial proteins and further supports the observation of a highly active metabolic state of infective larvae. The InterPro analysis allowed a further classification of the identified proteins, including those uncharacterized, either into families or on the basis of the presence of specific domains within their amino acid sequence. The most frequent InterPro domain and family entries are reported in Fig. 3, while the entire annotation is available as Additional file 1: Table S1. The CAP domain, SCP domain and thioredoxin domain were the most commonly represented protein domains, while several proteins were annotated as belonging to cysteine-rich secretory, transthyretin-like or peptidase protein families, making them the most represented protein families in the iL3 proteome. Overall, our functional analysis provides experimental evidence that confirms previous data on the most represented proteins associated with *S. stercoralis* parasitism, as inferred from genomic and transcriptomic data [11, 33, 37, 38], as well as with proteomics analyses of *S. ratti* and *S. venezuelensis* iL3 ESP [14, 17]. In particular, in our dataset we identified a high proportion of proteins with peptidase activity; such proteins have already been highlighted as potentially involved in parasitism as they are upregulated in the adult parasitic female stage of *S. ratti* and *S. stercoralis* [11]. Indeed, these proteins, including metalloproteases and metallopeptidases (such as astacin-like proteins), are involved in tissue degradation. This is a fundamental process in the initial phases of the infection for the penetration of host tissues and in parasite migration through the host body—even though peptidases could also contribute to immune evasion [39]. Other protein categories known to be associated with *S. stercoralis* parasitism and identified in our study include: (i) galectins, involved in pathogen adhesion to the host cells and activation of host innate and adaptive immunity [40]; (ii) transthyretin-like proteins; and (iii) SCP/TAPS-/CAP-domain containing proteins, with putative immunomodulatory properties in parasitic nematodes [41]. The expansion of SCP/TAPS coding genes in *Strongyloides* and *Parasitrongyloides* compared to *Rabditophanes* suggests that their gene products might be associated with human parasitism [11]. In our study, we identified 11 proteins either as SCP domain-containing proteins (*n* = 7) or as uncharacterized proteins containing the SCP-domain according to the InterPro analysis (IPR034113). Most of the protein categories that had already been proposed as associated with iL3 parasitism were thus experimentally confirmed in our proteomics study of *S. stercoralis* iL3 with 43 protein matches (Additional file 2: Table S2). It is worth noting that almost 50% of these proteins were uncharacterized and were assigned to those categories only following the GO and InterPro semi-automated annotation. The importance of focussing ‘omics studies not only on known and characterized genes and proteins, but especially on those “novel” or uncharacterized ones was already highlighted more than 10 years ago. Such an approach can achieve a more in depth knowledge of the molecular mechanisms associated with pathology but also with pathogen development [42], especially for organisms whose genome and proteome are not fully annotated, such as *S. stercoralis.*

The objective of our study was not only to improve our knowledge of the iL3 proteome with novel experimental evidence, but also to predict—among those identified from a clinical isolate—potential immunogenic proteins that could be useful for the development of novel serological tests for the accurate diagnosis of human strongyloidiasis or as vaccine candidates, as potentially recognized by antibodies present in patients’ serum.

A few studies dating back to the 1990s reported the investigation of the humoral immune response associated with the intensity of infection and the detection of immunoreactive iL3 polypeptides [43–45]. However, only a couple of studies applied immuno-proteomics MS/MS-based approaches to also identify the immunogenic proteins recognized by antibodies from infected subjects [13, 46]. Indeed, Rodpai and colleagues confirmed by immunoblotting the high frequency of some protein bands that had previously been reported as immunoreactive [43–45, 47], but also identified them by tandem MS based on protein homology with *S. ratti* [46]. In particular, they identified a 26-kDa band corresponding to 14–3-3 protein and a 29-kDa band corresponding to ADP/ATP translocase 4. Additional antigenic proteins were further identified by the same group after they had improved sample separation through two-dimensional gel electrophoresis prior to immunoblotting [13]; the majority of these proteins were also identified in the present study.

In silico approaches can be used as an alternative, or a complement, to the experimental identification of immuno-reactive proteins. The advent of immunoinformatics has actually led to the development of a number of tools that can assist researchers in B-cell epitope prediction. Moreover, it has been shown that using multiple prediction methods results in a more accurate epitope prediction than using individual tools [28, 48]. In agreement with this, in the present study we combined the use of a machine learning-based algorithm (i.e. BepiPred-2.0 [29]) and the evaluation of several physicochemical residue properties to predict linear B-cell epitopes. In order to avoid the selection of proteins highly conserved across helminths or similar to human ones, we first excluded all those having high homology, as established by our BLASTp analysis (Additional file 3: Table S3). Among the 29 proteins showing limited homology with *Homo sapiens* or other pathogens of clinical relevance, we selected 10 for use in the prediction of the presence of B-cell epitopes (Table 1). This selection was based on the following criteria: (i) proteins already highlighted as potentially associated with *S. stercoralis* parasitism [11, 33] or as immunogenic [13]; (ii) extracellular or plasma membrane proteins as per CC GO terms; (iii) proteins with peptidase activity according to the MF GO terms; and (iv) proteins associated with relevant InterPro domain, family or homologous superfamily (namely transthyretin-like domain, CAP domain, galectin, cysteine-rich, peptidase, protease inhibitors). According to UniProt, 60% of the selected proteins are already characterized, while the remaining 40% are still uncharacterized (Table 1). The six characterized proteins included three SCP domain-containing proteins (ACs: A0A0K0E6J0, A0A0K0EG68, A0A0K0DTP5), galectin (AC: A0A0K0ECK4), NTR domain-containing protein (AC: A0A0K0EMX1) and L3NieAg.01 (AC: Q9UA16 also known as Ss-NIE). It is worth noting that galectins have already been reported to be involved in host–pathogen interaction and to display immuno-regulatory properties in *S. ratti* [49]. Also, several commercial and in-house assays already use the recombinant Ss-NIE for *S. stercoralis* serodiagnosis [9, 50–53]. Ss-NIE is also included in a commercial research use only (RUO) serological test, together with Ss-IR, which was not identified in our dataset [9]. The presence of Ss-NIE within our selection further supports the validity of our approach. In our study we also identified most of the proteins already highlighted as potentially immunogenic by Rodpai and colleagues [13, 46]. However, since these proteins displayed high homology with either human or other related pathogen proteins, their analysis for epitope prediction was not pursued (Additional file 2: Table S2, Additional file 3: Table S3).

**Table 1:** List of potentially immunogenic proteins and the predicted B-cell epitopes

Protein AC	Protein name	e-value^a^ vs *Homo sapiens*	e-value^a^ vs other pathogens^b^	Protein properties relevant for selection	Epitope sequence
A0A0K0E6J0	SCP domain-containing protein	7.00E−24	Minimum 2E−25	Extracellular region [GO:0005576]	^105^VTQPPRPTARPFSRNPE
			Maximum 5.5	Integral component of membrane [GO:0016021]	^128^KPAPRPTIPPKTAKPG
				IPR014044: CAP domain	^147^APPNNRIDPMYIPNPDE
A0A0K0DY51	Uncharacterized protein	3.00E−06	Minimum 3E−11	IPR035940: CAP superfamily [11]	^16^ESKNEEVHPT
			Maximum 7.7	IPR018244: Allergen V5/Tpx-1-related, conserved site [11]	^40^AVEPPAETPAE
					^72^PVETTTETP
					^140^PVETPAETSVDAPTENPTEVSADVPSTE
					^186^SVPEQSVEKIEEPSVTEVQCP
A0A0K0ECK4	Galectin	1.00E−13	Minimum 1E−22	IPR001079: Galectin, carbohydrate recognition domain	^10^TYDHIPKESYSIQPR
			Maximum 7.5		^91^KWQHEERTPKGNPFK
A0A0K0EG68	SCP domain-containing protein	7.00E−10	Minimum 2E−11	IPR001283: Cysteine-rich secretory protein-related	^66^RPTNRPINKKPIKKPNNKPK
			Maximum 1.8	IPR014044: CAP domain [11]	^105^PKPPGPRPKPPG
					^123^GPRPKPPG
					^137^GPKPKPTTTKPKPKPTTTKPKPKPTTTKPKPTQPPT
A0A0K0EMX1	NTR domain-containing protein	3.00E−05	Minimum 7E−15	Extracellular region [GO:0005576]	^125^MSPEKSPRYIYPPE
			Maximum 8.0	IPR001820: Protease inhibitor I35 (TIMP) [11]	^145^EVKNNLRTN
Q9UA16	L3NieAg.01 (Fragment)	4.00E−25	Minimum 2E−30	Extracellular region [GO:0005576]	^75^YNYDNDKA
			Maximum 4.0	IPR014044: CAP domain [11]	^131^LEHDPKNRIE
A0A0K0E2F4	Uncharacterized protein	1.00E + 00	Minimum 1.1E−02	Integral component of membrane [GO:0016021]	^40^IDNQPAYV
			Maximum 4.1	IPR007863: Peptidase M16, C-terminal [11]	^75^HKIPHEPKASAREGVDGDEEDGASDTF
A0A0K0DTP5	SCP domain-containing protein	1.00E−13	Minimum 4E−16	IPR014044: CAP domain [11]	^108^KQHNYDRDT
			Maximum 5.9		
A0A0K0ELA9	Uncharacterized protein	2.90E−01	Minimum 4E−13	Integral component of membrane [GO:0016021]	^44^FGKKDFSTKDLEPKNLKD
			Maximum 8.6	IPR001534: Transthyretin-like [11]	
A0A0K0E132	Uncharacterized protein	8.40E−01	Minimum 2E−19	Integral component of membrane [GO:0016021]	No B-cell epitope found
			Maximum7.3	IPR001534: Transthyretin-like [11]	

^a^e-value from BLASTp search

^b^The largest and smaller e-value from the homology analysis with other pathogens are reported. Other pathogens include:* Ancylostoma duodenale*;* Ancylostoma ceylanicum*;* Necator americanus*;* Ascaris lumbricoides*;* Trichuris trichiura*;* Toxocara canis*;* Loa loa*;* Mansonella perstans*;* Mansonella ozzardi*;* Wuchereria bancrofti*;* Onchocerca volvulus*;* Brugia malayi*;* Brugia timori*;* Dirofilaria immitis*;* Dirofilaria repens*;* Trichinella spiralis*;* Taenia saginata*;* Taenia solium*;* Echinococcus granulosus*;* Hymenolepis nana*;* Schistosoma mansoni*;* Schistosoma haematobium*;* Schistosoma japonicum*;* Fasciola hepatica*;* Plasmodium falciparum*;* Plasmodium vivax*;* Plasmodium ovale*;* Plasmodium malariae*;* Plasmodium knowlesi*.

**Fig. 4:**
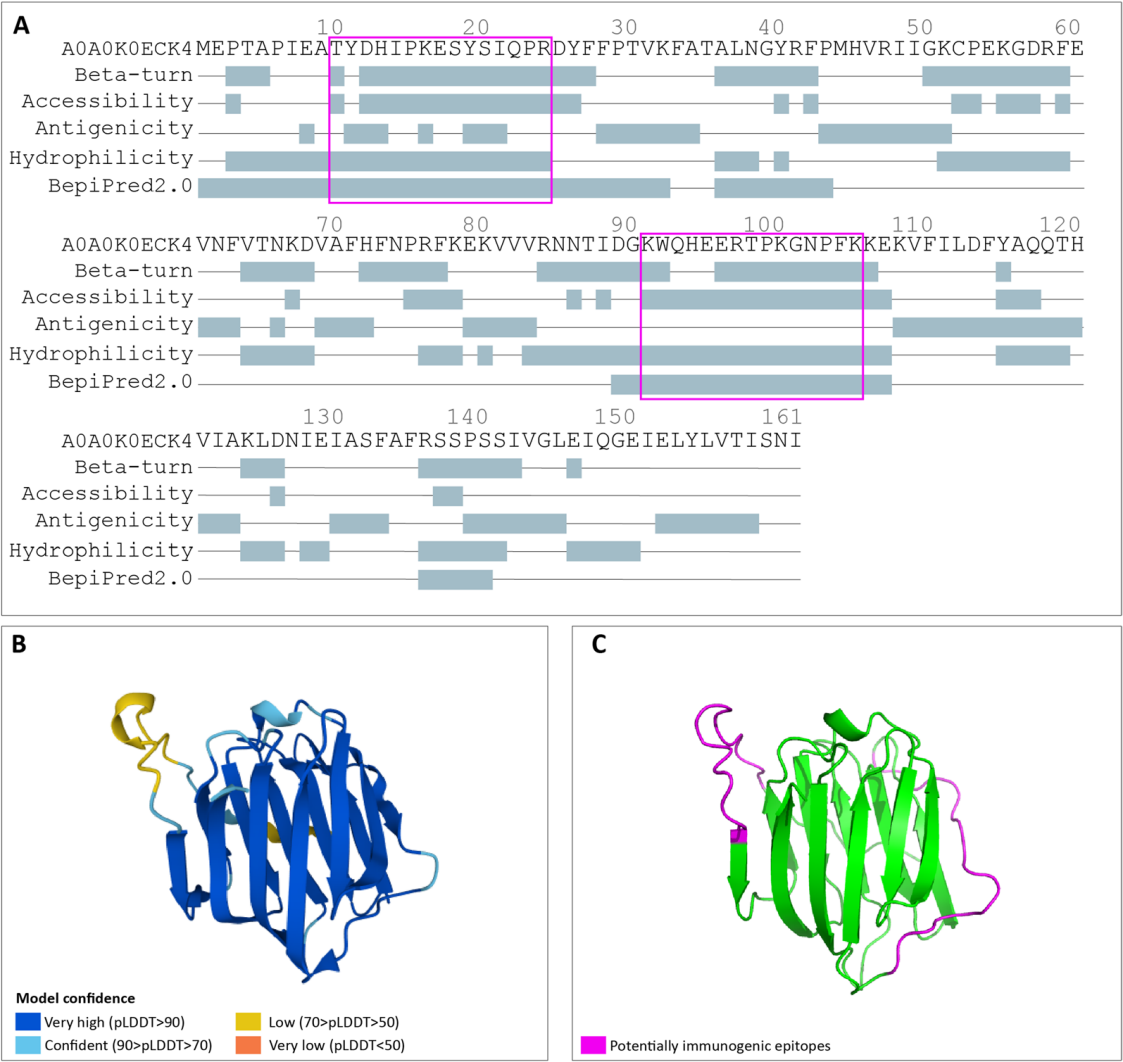
B-cell epitope prediction results. The results for the protein A0A0K0ECK4—galectin are reported as an example. **a** FASTA sequence showing the results obtained with each tool (Chou & Fasman Beta-Turn Prediction; Emini Surface Accessibility Prediction; Kolaskar & Tongaonkar Antigenicity; Parker Hydrophilicity Prediction, BepiPred2.0; all available via http://tools.iedb.org/bcell/). All residues having a score above their threshold are highlighted in grey. The purple squares indicate the sequences highlighted as being potentially immunogenic as reported in the Methods section. **b** Protein structures as predicted by AlphaFold showing the model confidence. **c** Mapping of the potentially immunogenic epitopes on the protein structure. The same images for all other selected proteins are reported in Additional file 4: Figures S1–S8

The B-cell epitope prediction highlighted that nine out of the 10 selected proteins contained epitopes with high consensus across the different tools employed (Table 1; Fig. 4; Additional file 4: Figures S1–S8); these were therefore considered as potentially immunogenic. The remaining protein (AC A0A0K0E132, uncharacterized protein) did not display potentially immunogenic epitopes as per our analysis. The structural models, together with a confidence estimation as per AlphaFold, are reported in Fig. 4 and Additional file 4: Figure S1–S8. In agreement with the results obtained from different web-based prediction tools, all epitopes were exposed to the external environment, thus potentially accessible for antibody binding. However, some epitopes fell within regions of the structure which was modelled with low confidence. This could be explained by the fact that immunogenic epitopes often fall within highly variable regions, and there is a lower confidence in the structure as predicted by AlphaFold.

A recent work employed a reverse in silico approach to predict immunogenic proteins from the *S. stercoralis* proteome available in UniProt [54]. However, none of the proteins proposed as potentially immunogenic was identified in our dataset, probably because the analysis was performed on the entire *S. stercoralis* proteome, without taking into account the parasite developmental stage.

In the present study we did not perform a comparison of protein expression between larval developmental stages, as has been done at the transcriptomic level or for other *Strongyloides* species [11, 14, 33, 34, 38], thus we cannot speculate on the role of iL3 proteins in larval development. However, a comparison using quantitative proteomics of different larval stages might contribute to corroborate these transcriptomics data and might identify novel proteins potentially involved in parasitism and/or in parasite development that could be of interest for the development of novel disease control strategies. Similarly, investigations should be extended to the study of ESPs released from *S. stercoralis* iL3, as has already done for *S. ratti* [14] and *S. venezuelensis* [17], as these could highlight additional candidates for serodiagnosis.

Proteomics data on *S. stercoralis* are still limited, and the reference database and proteome are in continuous evolution. Therefore, some proteins ID here reported might change in the future.

## Conclusions

In conclusion, we provide the largest experimental dataset of the *S. stercoralis* iL3 proteome. By presenting, for the first time, an extensive proteomics dataset from the analysis of iL3 isolated from a clinical sample, our study brings knowledge on the *S. stercoralis* proteome to a level comparable to our knowledge on its close relatives *S. ratti* and *S. venezuelensis* [55]. These data may be useful for future studies as they represent a step towards filling the current gap in experimental proteomics data. Indeed, a broader expertise about protein expression in *S. stercoralis* larvae, as well as their modulation during different developmental stages, will be essential for identifying novel therapeutic and vaccine targets.

Our semi-automated annotation allowed us to confirm the presence—at the proteome level—of protein categories potentially involved in parasitism that to date were only inferred from genomics and transcriptomics data. Moreover, additional protein groups deserving further investigation, such as oxidoreductases, were also highlighted. Finally, we also propose a number of immunogenic protein candidates that, if experimentally confirmed, might be considered in the future for the development of novel serological diagnostic tests that could make the diagnosis of this neglected tropical disease more reliable and accurate.

## Supplementary Information

**Additional file 1: Table S1.** Annotated dataset. The dataset includes peptide list, protein identification, gene ontology annotation and InterPro annotation.

**Additional file 2 : Table S2.** Proteins identified in the present study and already reported in the literature as: (i) associated with* Strongyloides *parasitism; (ii) part of iL3 proteome; (iii) potentially immunogenic.

**Additional file 3 : Table S3.** Homology with* Homo sapiens* and other pathogens of clinical importance as potentially responsible for co-infections with* S. stercoralis*.

**Additional file 4: Figures S1–S8.** B-cell epitope prediction results. For each figure:** a** FASTA sequence showing the results obtained with each tool (Chou & Fasman Beta-Turn Prediction; Emini Surface Accessibility Prediction; Kolaskar & Tongaonkar Antigenicity; Parker Hydrophilicity Prediction, BepiPred2.0; all available via http://tools.iedb.org/bcell/). All residues having a score above their threshold are highlighted in grey. The purple squares indicate the sequences highlighted as potentially immunogenic, as reported in the Methods section. For each figure: ** b** Protein structures as predicted by AlphaFold showing the model confidence;** c** mapping of the potentially immunogenic epitopes on the protein structure.

## Data Availability

The mass spectrometry proteomics data have been deposited to the ProteomeXchange Consortium via the PRIDE partner repository with the dataset identifier PXD037243.

## Abbreviations

BP: Biological process
CC: Cellular component
ESP: Excretory-secretory product
GO: Gene ontology
iL3: Infective filariform larvae
L1: Rhabditiform larva
LC–MS/MS: Liquid chromatography-tandem mass spectrometry
MF: Molecular function
PBS: Phosphate buffered saline
ROS: Reactive oxygen species
