# Supplementary material for: Novel insights into the somatic proteome of Strongyloides stercoralis infective third-stage larvae
Source: Parasit Vectors. 2023 Jan 31;16:45. doi: 10.1186/s13071-023-05675-7 (PMC9890704; doi:10.1186/s13071-023-05675-7)

Novel insights into the somatic proteome of *Strongyloides stercoralis* infective third-stage larvae

Klevia Dishnica[1], Chiara Piubelli[2], Marcello Manfredi[3], Ravi Teja Kondaveeti[2], Silvia Stefania Longoni[2], Monica Degani[2], Dora Buonfrate[2], Alejandro Giorgetti[1], Natalia Tiberti[2]

1 Department of Biotechnology, University of Verona, Verona (Italy)

2 Department of Infectious, Tropical Diseases and Microbiology, IRCCS Sacro Cuore Don Calabria Hospital, Negrar di Valpolicella (Italy)

3 Department of Translational Medicine, University of Piemonte Orientale, Novara (Italy)

Supporting Figures S1-S8. B-cell epitope prediction results. For each figure: **A)** FASTA sequence showing the results obtained with each tool (Chou & Fasman Beta-Turn Prediction; Emini Surface Accessibility Prediction; Kolaskar & Tongaonkar Antigenicity; Parker Hydrophilicity Prediction, BepiPred2.0; all available via http://tools.iedb.org/bcell/); all residues having a score above their threshold are highlighted in grey. The purple squares indicate the sequences highlighted as potentially immunogenic as reported in the methods section. **B)** Protein structures as predicted by AlphaFold showing the model confidence. **C)** Mapping of the potentially immunogenic epitopes on the protein structure.

Figure S1. B-cell epitope prediction results for the protein A0A0K0E6J0 - SCP domain-containing protein.

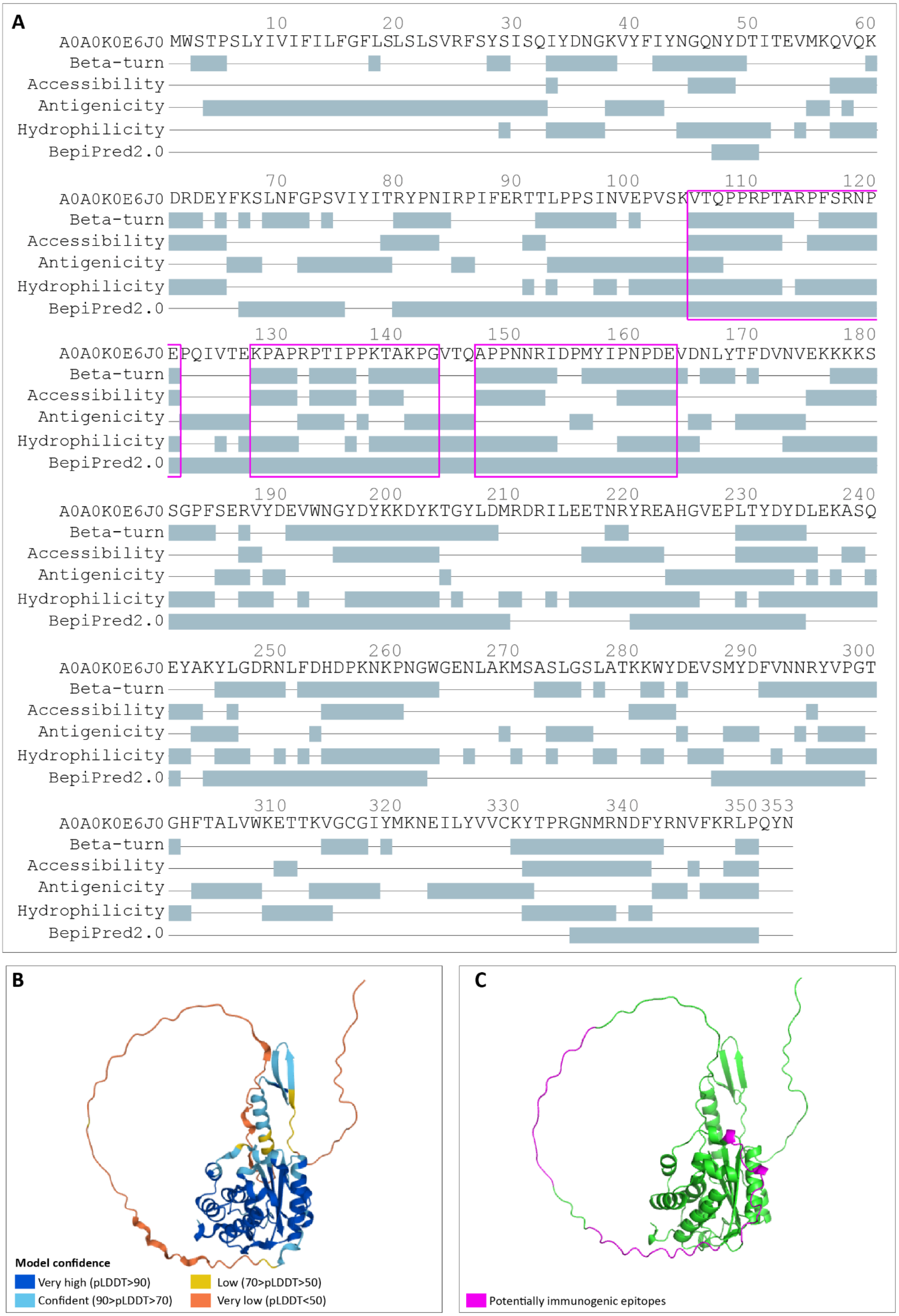
A
A0A0K0E6J0 MWSTPSLYIVIFILFGFLSLSLSVRFSYSISQIYDNGKVYFIYNGQNYDTITEVMKQVQK
A0A0K0E6J0 DRDEYFKSLNFGPSVIYITRYPNIRPIFERTTLPPSINVEPVSKVTQPPRPTARPFSRNP
A0A0K0E6J0 EPQIVTEKPAPRPTIPPKTAKPGVTQAPPNNRIDPMYIPNPDEVDNLYTFDVNVEKKKKS
A0A0K0E6J0 SGPFSERVYDEVWNGYDYKKDYKTGYLDMRDRILEETNRYREAHGVEPLTYDYDLEKASQ
A0A0K0E6J0 EYAKYLGDRNLFDHDPKNKPNGWGENLAKMSASLGSLATKKWYDEVSMYDFVNNRYVPGT
A0A0K0E6J0 GHFTALVWKETTKVGCGIYMKNEILYVVCKYTPRGNMRNDFYRNVFKRLPQYN
Beta-turn
Accessibility
Antigenicity
Hydrophilicity
BepiPred2.0
B
Model confidence
Very high (pLDDT>90)
Confident (90>pLDDT>70)
Low (70>pLDDT>50)
Very low (pLDDT<50)
C
Potentially immunogenic epitopes

Figure S2. B-cell epitope prediction results for the protein A0A0K0DY51 - Uncharacterized protein.

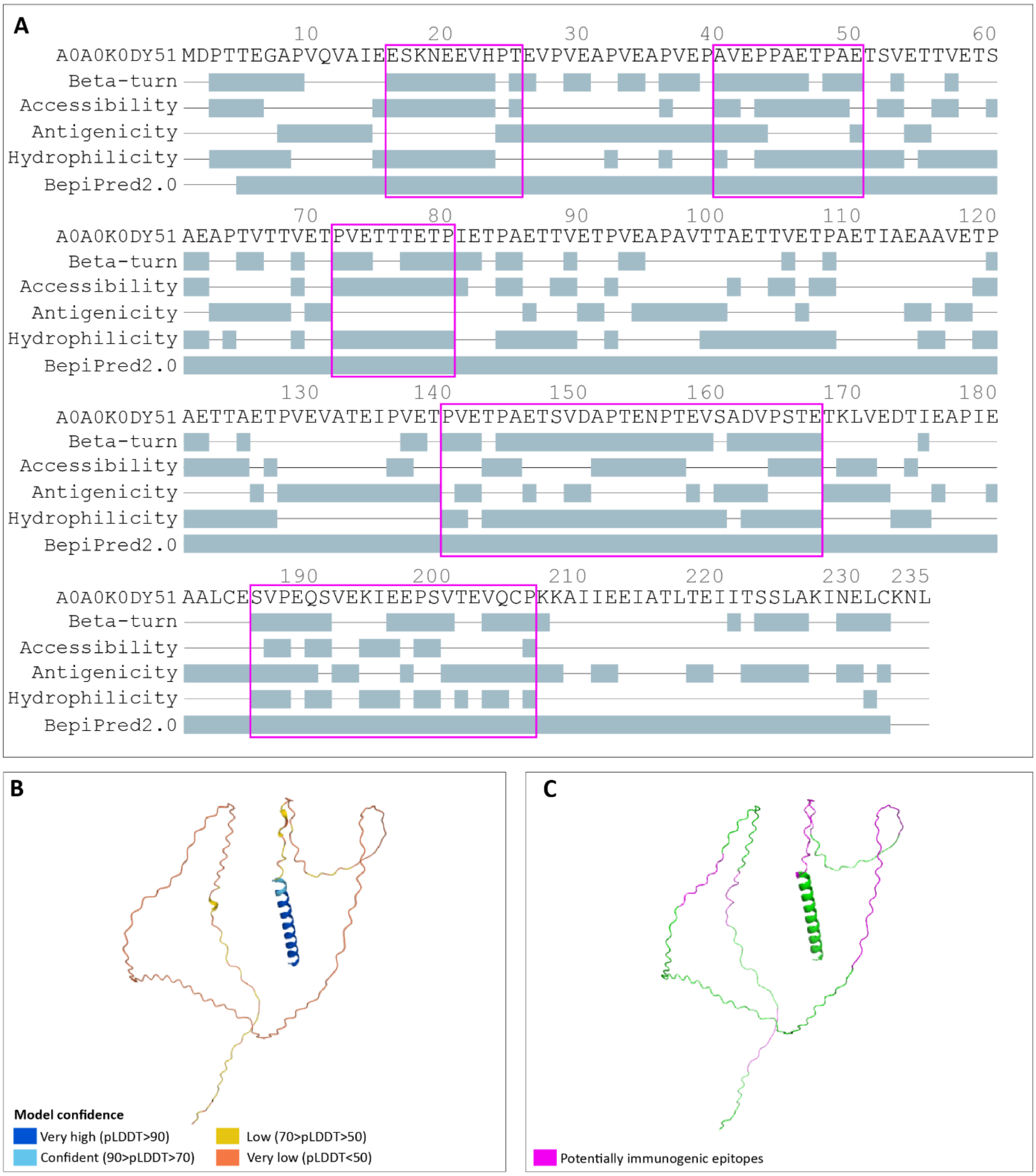

Figure S3. B-cell epitope prediction results for the protein A0A0K0EG68 - SCP domain-containing protein.

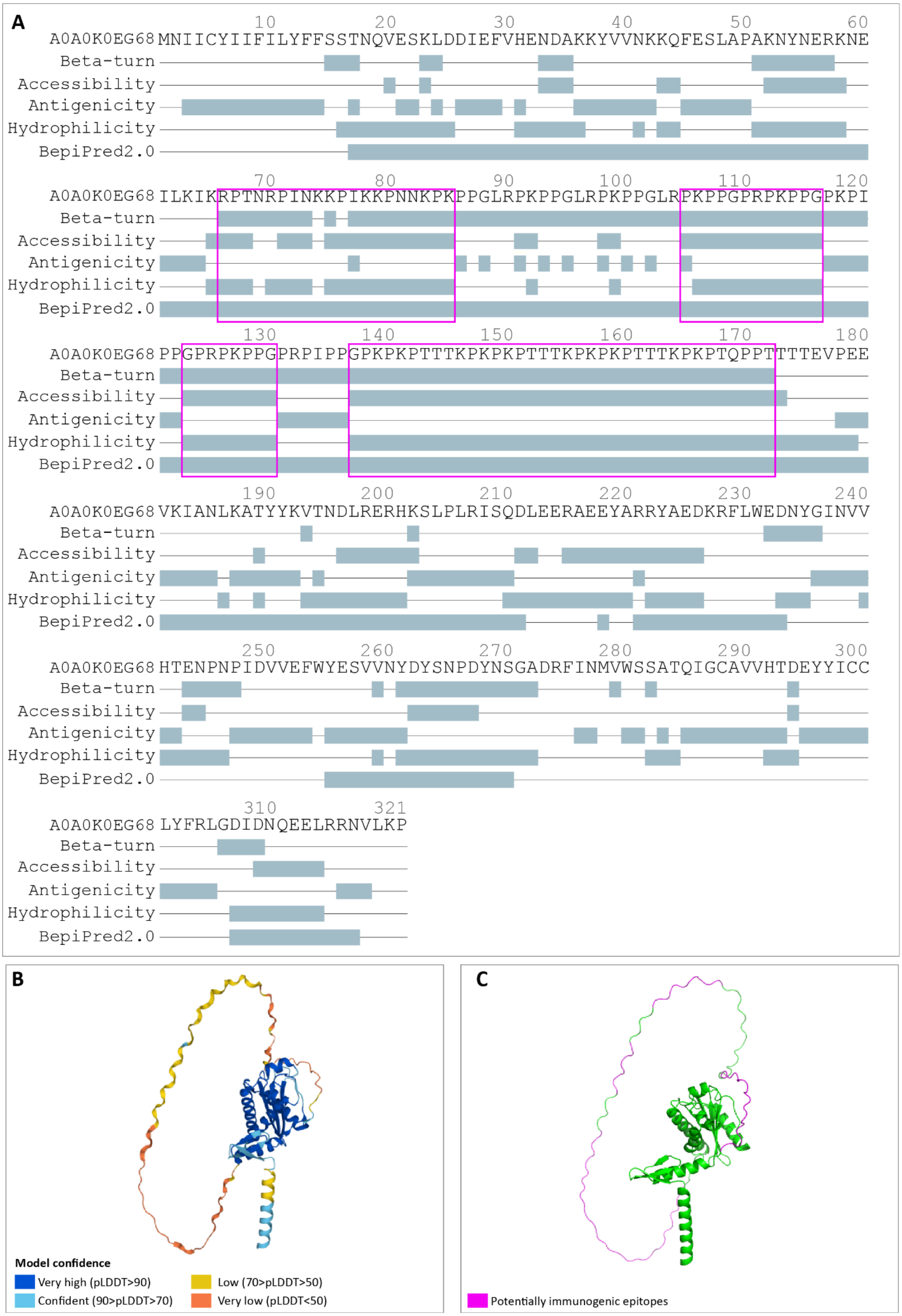

Figure S4. B-cell epitope prediction results for the protein A0A0K0EMX1 - NTR domain-containing protein.

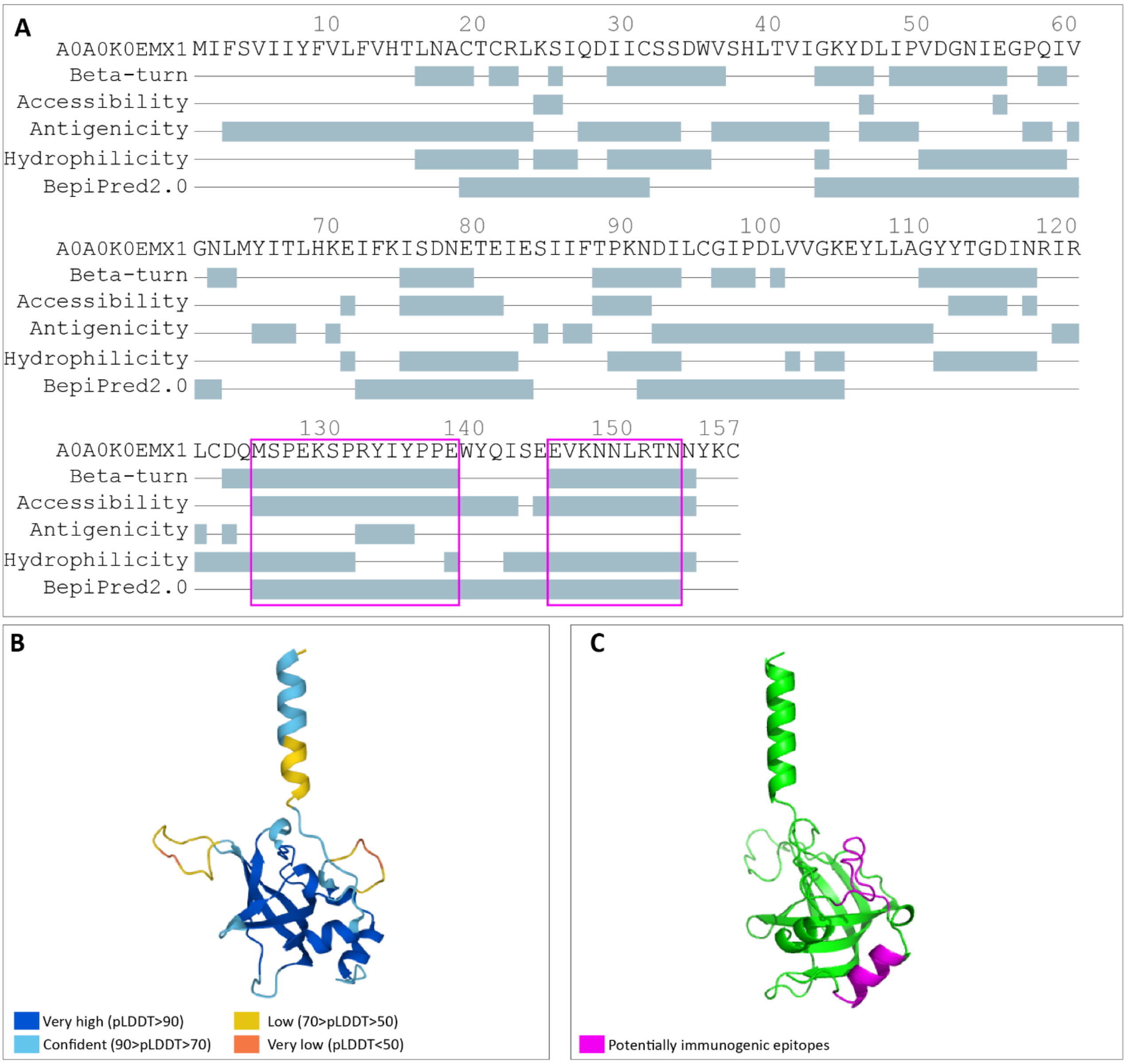

Figure S5. B-cell epitope prediction results for the protein Q9UA16 - L3NieAg.01.

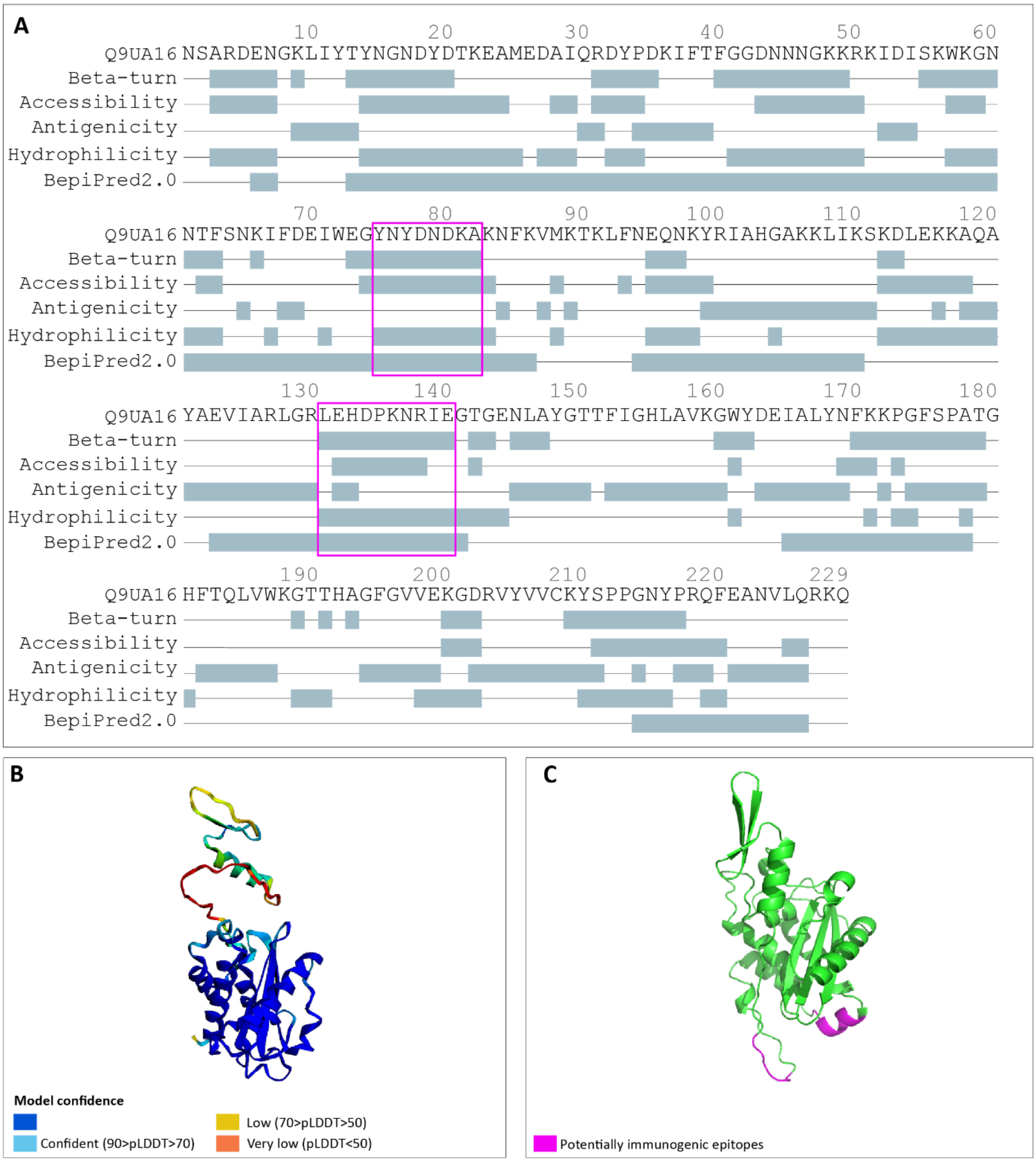

Figure S6. B-cell epitope prediction results for the protein A0A0K0E2F4 - Uncharacterized protein.

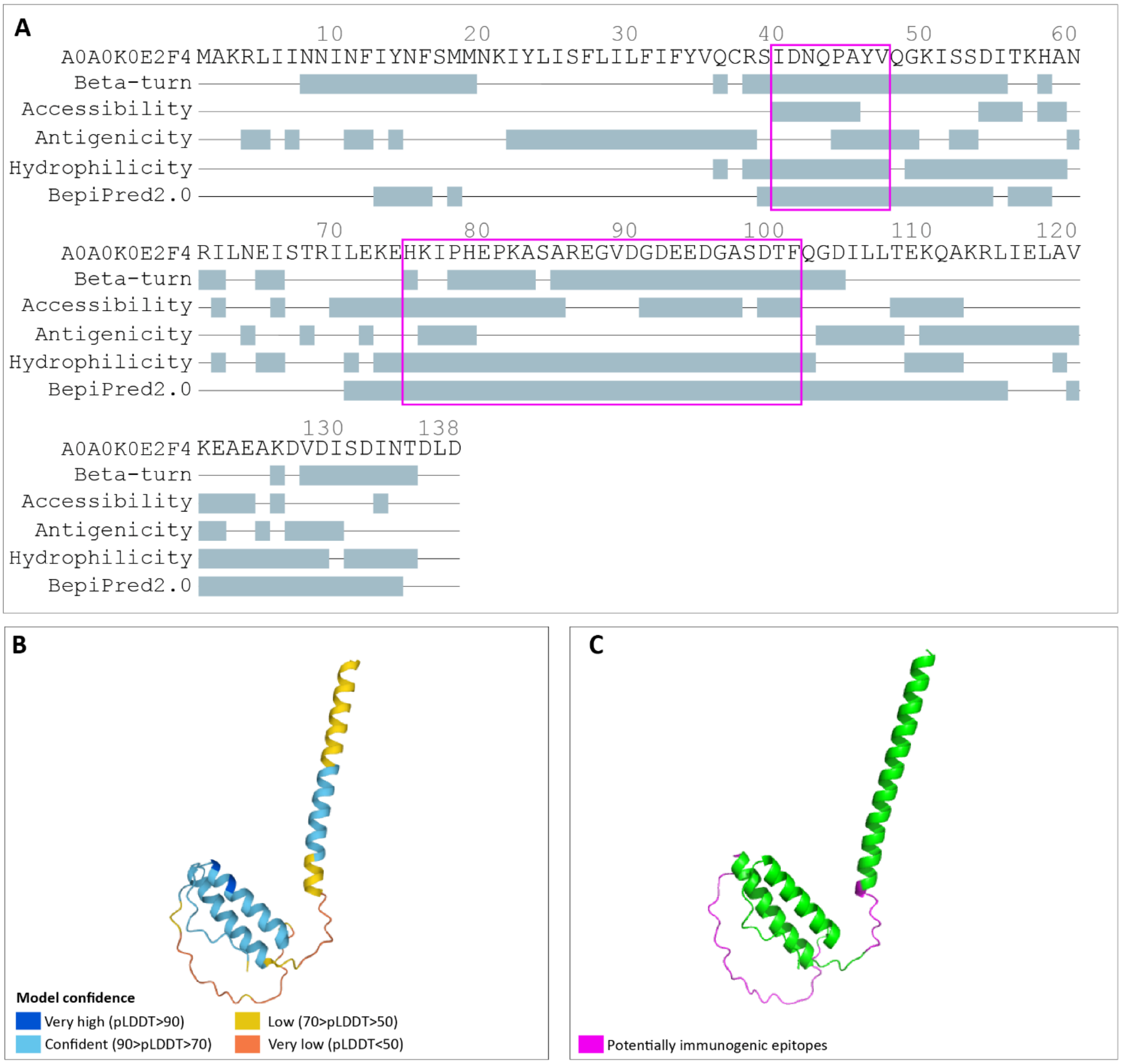

Figure S7. B-cell epitope prediction results for the protein A0A0K0DTP5 - SCP domain-containing protein.

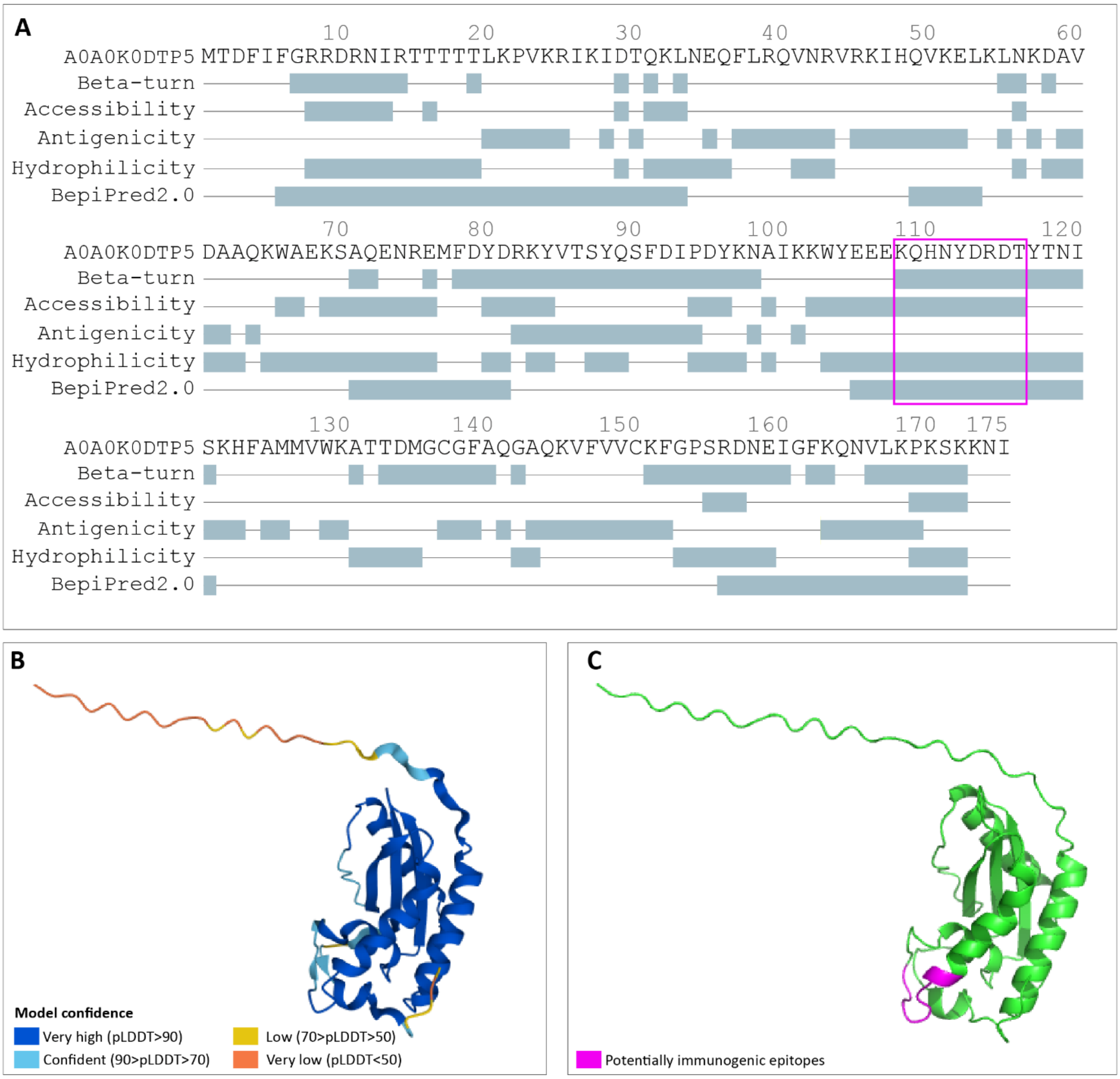

Figure S8. B-cell epitope prediction results for the protein A0A0K0ELA9 - Uncharacterized protein.

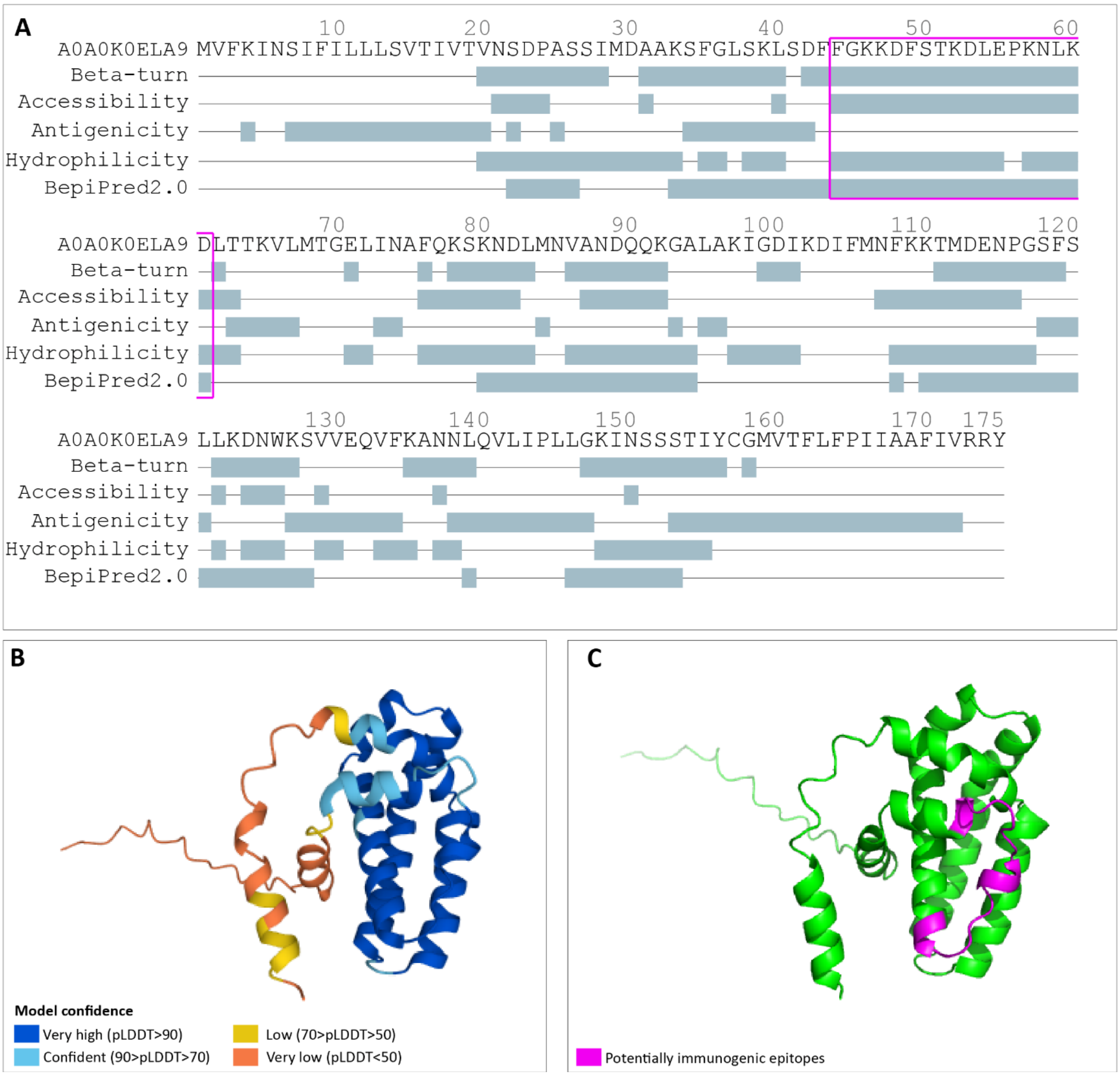

Supplement: Supplementary file 4 — Additional file 4: Figures S1–S8. B-cell epitope prediction results. For each figure: a FASTA sequence showing the results obtained with each tool (Chou & Fasman Beta-Turn Prediction; Emini Surface Accessibility Prediction; Kolaskar & Tongaonkar Antigenicity; Parker Hydrophilicity Prediction, BepiPred2.0; all available via http://tools.iedb.org/bcell/). All residues having a score above their threshold are highlighted in grey. The purple squares indicate the sequences highlighted as potentially immunogenic, as reported in the Methods section. For each figure: b Protein structures as predicted by AlphaFold showing the model confidence; c mapping of the potentially immunogenic epitopes on the protein structure. [file 13071_2023_5675_MOESM4_ESM.pdf]
